# Transition From a Vulnerable Community to Resilient One in Bauchi State, Nigeria

**DOI:** 10.5334/aogh.4741

**Published:** 2025-09-30

**Authors:** Umar Ibrahim, Georgina Yeboah, Judy Khanyola

**Affiliations:** 1 Public and Environmental Health Department, Federal University Dutse, Jigawa State, Nigeria; 2 African Forum for Research and Education in Health, AFREhealth Secretariat, School of Public Health, Kwame Nkrumah University of Science and Technology, Kumasi, Ghana

**Keywords:** adaptation, community, climate, health, resilience

## Abstract

*Background:* The Kulawa village, a hard-to-reach community in Bauchi State, Nigeria, was visited by an NGO initially for mass HIV and malaria screenings. However, it was discovered that women remained isolated at home despite facing maternal health challenges such as prolonged labor and other pregnancy complications. These issues were worsened by climate change, particularly during annual floods, which created severe barriers to accessing healthcare, leaving the community dependent on traditional birth attendants (TBAs) and distant health centers.

*Objective:* The case study aims to present health access challenges exacerbated by climate change and the implementation of sustainable local healthcare workforce strategies that improve maternal healthcare adaptation in the Kulawa community.

*Methods:* The NGO engaged community stakeholders to discuss the health impacts of climate change, especially during flooding and emphasized early health-seeking behaviors. The community supported the course by donating land where the community clinic was built. The NGO also initiated a community education program, identifying 15 young children (ages 13–15) and providing them with the resources needed to pursue healthcare education, with the long-term goal of creating a sustainable local health workforce.

*Findings:* Fifteen children aged 13–15 were supported with bicycles, books, and extra lessons, eventually passed their high school examinations, and proceeded to medical and health professional training. Fourteen graduated from healthcare professions and were initially engaged in voluntary services and eventually employed by the Bauchi Local Government Health Authority. Their deployment to their community significantly improved health outcomes.

*Discussion:* This case signifies the role of climate change adaptation in healthcare. The Kulawa community transformed from traditional practices to a resilient health system through education, community engagement, and capacity building. The initiative highlights the effectiveness of culturally sensitive, community-driven approaches in addressing health risks posed by climate change.

## Background

**Figure 1:**
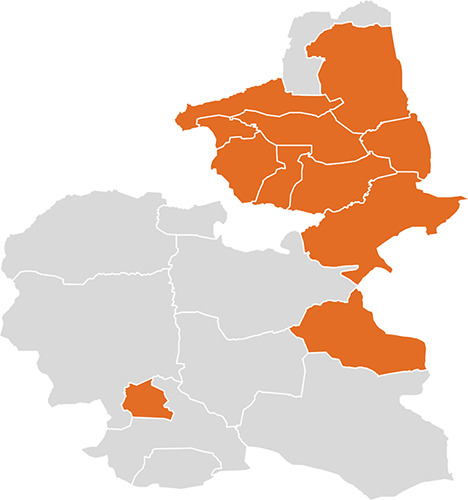
A MAP Showing Locations With High Maternal Health Challenges in Bauchi State, Nigeria.

Bauchi State, Nigeria, experiences significant environmental stressors linked to climate change, including rising temperatures, unpredictable rainfall, and prolonged droughts. These extreme weather events worsen food insecurity and water scarcity while also increasing the frequency of vector-borne diseases such as malaria. The compounded impacts of these stressors lead to heightened disease transmission, malnutrition, and strain on an already overstretched healthcare system, with particularly tragic consequences for pregnant women. The region’s climate conditions also exacerbate the vulnerability of women in hard-to-reach rural areas, leading to poor maternal and child health outcomes, malnutrition, and mental health challenges. This underscores the urgent need for climate adaptation strategies, with case studies offering insights into effective interventions (Figure 1).

The case study was conducted in Bauchi Local Government Area (LGA), Bauchi state, Nigeria, West Africa. Bauchi state is situated between latitudes 9° 3’ and 12° 3’ north and longitudes 8° 50’ and 11° east. The state has a population of over 4.6 million, covering an area of 49,119 km^2^ (18,965 sq mi), or about 5.3% of Nigeria’s land area. Islam is the predominant religion, and Hausa and Fulani are the predominant languages. A total of 73% of women in the state and 38% countrywide were reported as having no formal (Western) education. The main source of income in the state is peasant agriculture (66%), with a typical family size of 7–8 people per household and a minimum of 9 people in an extended family’s home [1–3].

The state is susceptible to environmental deterioration due to man-made and natural factors. As such, climate change effects on health outcomes in the state are linked to complex anthropogenic factors of emissions from fossil fuels and unsustainable land use for agriculture and deforestation, with resultant consequences of desertification, pollution, flooding, and erosion, among others [4, 5]. As explained above, vulnerable populations such as pregnant mothers are affected the most in the case study area.

The case study was based on a community engagement technique utilized in a hard-to-reach community. The community used to experience a high rate of pregnancy-related deaths due to annual flooding that makes accessing antenatal care (ANC) services difficult. What was achieved in that community was through a community engagement approach to controlling flood-related maternal mortality rates. The journey began when a local NGO visited the community in 2014, where interaction was made with women through community leadership; the women complained of miscarriages and deaths during annual flood events. The pregnant women had ANC services at a government health facility, located about 20 km away from their village, and accessing it included a riverine crossing, which made it unreachable during the annual episode of floods. To address the looming challenge, the community was advised to train its members to become health personnel and also construct a community-owned health center so that on completion of the training, they can return home to serve their community. By adapting these strategies, the community was able to address its annual challenges. Other approaches used include behavior change communication, building a climate change resilience health system, and reducing exposures to vulnerable pathways, such as flooding.

Floods have become an annual routine event over the years in that community. Whenever it rains, the severity of the precipitation produces floods and soil erosion, frequently resulting in fatalities and extensive damage to houses, clinics, and road networks. This causes inaccessibility to healthcare services, especially for pregnant women in hard-to-reach communities, seasonally due to these climate-related issues. It was predicted that Bauchi state would be at risk of flooding and other effects of environmental change, such as maternal-related deaths [1, 4].

The challenge was addressed through community engagement. To achieve this, a convincing life video clip on climate change effects, with high related risks, was shown to the community. After watching the video content, approval was granted by the men through the community leadership, to discuss with the women at the household level, an opportunity that was always denied before the video display. Information on the maternal health challenges the women encountered in the village was obtained, and the obtained data gave birth to the adaptation techniques currently enjoyed by the community.

Indeed, community engagement is among the viable options for adapting to climate change’s effects on health. When it comes to adaptation to the dangers of floods in maternal-related mortalities, community engagement proved to be a viable technique. Community engagement guarantees that adaptation techniques are context-specific and culturally acceptable while also fostering a sense of ownership among local populations involved in the development and execution of flood adaptation measures. The strategy allows the communities to be actively involved in the design of approaches that address the needs of expectant mothers during the annual episodes through improved healthcare infrastructure and personnel. Therefore, community engagement ensures resilience, lessens the vulnerability of maternal health during flood events, and eventually lowers the rates of maternal death linked to climate-induced disasters by fusing local solutions with scientific understanding.

## Approach

Previous studies in Bauchi State have established a clear connection between maternal mortality and environmental changes during pregnancy and childbirth [1, 6]. In response to these findings, a local NGO visited the Kulawa community to investigate the high maternal mortality rate and identify underlying causes. During the visit, the NGO made several recommendations to address the issue, which the community later adopted as part of their adaptation efforts. To assess the success of these interventions, a follow-up visit was conducted, building on the primary data collected earlier. This follow-up confirmed the community’s compliance with the recommendations and marked a significant step forward in their journey toward improving maternal health.

The follow-up assessment employed a range of qualitative research methods, including focus group interviews, community engagement, and participatory research. These approaches allowed for a deep exploration of the community’s progress, with data saturation achieved through repeated cross-checking of the initial and subsequent findings. To document the outcomes and provide replicable insights, the case study adopted a narrative approach, based on repeated visits to the community over nine years since the initial advocacy visit. This allowed for a comprehensive understanding of the community’s health adaptation journey in the face of climate-related challenges.

Key findings from the assessment revealed significant improvements in mothers’ attitudes toward utilizing ANC services. The community engagement strategies employed by the NGO proved highly effective in adapting to the threat of flooding, which had previously hindered pregnant women’s access to healthcare. The study identified and addressed three critical barriers: ignorance, lack of health services, and cultural isolation. These issues were mitigated by improving accessibility to healthcare, eliminating the need for dangerous river crossings, and enhancing awareness of the importance of ANC among expectant mothers.

The outcomes of the interventions provide valuable insights into how targeted strategies can improve maternal health outcomes, particularly in communities affected by climate change. By addressing the specific challenges posed by extreme weather events, the case study demonstrates the potential for community-driven solutions to mitigate health risks and ensure resilience. These lessons offer a blueprint for other hard-to-reach communities facing similar environmental stressors, helping them adapt to the growing challenges of climate change.

Looking ahead, this case study lays the groundwork for future research into local solutions for climate change-related health adaptations. The successful implementation of strategies in Kulawa highlights the importance of developing tailored interventions that consider both the environmental and cultural context, paving the way for more effective healthcare delivery in vulnerable regions.

## Findings

During the NGO’s visit to the Kulawa community, it was observed that women were largely confined indoors, interacting mainly with their relatives and fellow women. The initial agenda was to conduct mass HIV and malaria screenings, but interactions revealed tragic annual incidents linked to climate change that affected maternal health. Community members often relied on traditional birth attendants (TBAs) and health centers for ANC services. Women typically opted for traditional practices during normal pregnancies, seeking medical attention only when complications arose. Unfortunately, complications during the annual flooding often resulted in fatalities due to the inability to cross flooded streams.

The primary healthcare center was situated about 20 km away from the village. Seasonal floods made river crossings perilous, posing an annual challenge for pregnant women facing complications. Usually, complications developed after prolonged labor before the women were referred to the health center, often on the recommendation of TBAs. The main mode of transportation was motorcycles, which became impractical during floods. Consequently, makeshift bamboo stretchers were used to carry victims, but many could not reach the healthcare center in time, leading to deaths from prolonged labor, stress, and the distance to medical facilities. The NGO was shocked by these findings, which motivated them to explore solutions to help the community overcome these challenges.

## Discussion

In response to the accessibility challenges facing the community, the NGO facilitated discussions with local stakeholders, highlighting the difficulties in providing clinics, schools, and other essential infrastructure. The nearest school was also several kilometers away, which exacerbated the situation. Through consultations, the NGO emphasized the importance of early health-seeking behaviors, leading the community to agree on establishing health facilities and employing health workers from within.

Initially, the community expressed concerns about how to achieve these goals, as none of them were high school graduates, and very few children attended school. In response, the NGO outlined a self-reliance plan to create and maintain a sustainable health workforce through community education. This approach resonated with community members, who recognized the need for trained health personnel. As a result, committed and capable children were identified and sponsored for education in a nearby village.

The NGO identified 15 children aged 13–15, counseling them on the importance of healthcare services. They received bicycles for transportation, along with books, uniforms, and breakfast tokens. Extra lessons in English and mathematics were arranged to prepare them for careers in healthcare. Financial support for these initiatives was gathered through community contributions and external support from the NGO. After three years, all 15 students passed the Senior School Certificate Examination (SSCE), enabling them to pursue further education in nursing and community health.

The students were categorized based on their chosen fields: six girls attended the College of Midwifery, two boys went to the College of Nursing, and the remaining seven studied Community Health Extension Work at the College of Health Technology. Fourteen of the 15 students graduated and obtained professional registration, eventually securing employment with the Bauchi Local Government Health Authority, after voluntary services, with deployments to their own and neighboring communities. The contributions of these trained health professionals encouraged increased community participation in healthcare initiatives, and initial services were provided voluntarily. The clinics were sustained through cost-recovery practices, leading to the local government’s decision to support more volunteers to study health professional courses for eventual employment as health workers while constructing additional clinics in neighboring communities.

The community’s decision to support their children’s education and engage in community services has led to a transformative shift from adherence to taboos to a dynamic community with well-trained Indigenous health professionals. This case study underscores the significance of teamwork, education, and environmentally friendly practices in promoting health and well-being. The improvements in health outcomes—evidenced by lower morbidity and mortality rates and longer life spans—demonstrate the effectiveness of culturally sensitive, community-driven approaches to dismantle harmful attitudes and behaviors while enhancing overall health resilience in the face of climate change.

### Lessons learned

- Community ownership and active engagement are essential for a successful transition from a taboo community to a modern health adaptation one.
- Change in attitudes and behaviors is essential in establishing trust and promoting candid communication.
- It is easier for new health practices to be accepted when cultural norms are recognized and respected.
- Improving receptivity and sustainability is achieved by designing interventions based on cultural values.
- Community education and awareness initiatives provide the public with information that facilitates well-informed decision-making and encourages long-term behavioral modifications.
- The impact of interventions will increase by working together with local authorities, NGOs, and community members in addressing the identified climate issues.

### Challenges in formulating the case study

- The community’s early hostility and mistrust made it difficult to collect precise and thorough data from the beginning of the engagement.
- To accurately evaluate the effects of certain factors and separate them, complex modeling and analysis are necessary, especially when connecting health outcomes to climate change adaptation.
- The scope, speed, and efficiency of data gathering and implementation were hampered by a lack of funding and human resources.

### Effective adaptation technique

The adopted approach showcases the community’s transformation from experiencing regular seasonal maternal morbidity and mortality challenges linked to climate change to adaptation practices that mitigate these annual maternal threats. The approach addressed cultural and natural barriers through simple techniques that solved the associated hard-to-reach challenges confronting the community. Despite that, the collected data need further saturation on how the adapted technique by the community was successfully executed, and why other communities may have a challenge adopting a similar approach. Nevertheless, the approach can be adopted by other communities with similar challenges, because it was not tried elsewhere.

In this regard, the case study points out that a diverse scientific workforce with experience in public health, environmental studies, demography, and statistics is required to conduct effective adaptation research, due to its multidisciplinary facets. Therefore, developing the required capacity for adaptation research thrives on training programs that give researchers expertise in community-based participatory research and adaptive techniques. Additionally, building strong networks to support information sharing and collaborative research projects between disciplines and geographical areas, with support from funding bodies for long-term research projects, is essential.

### Future directions and recommendations

- Preserve the progress of community involvement using continuous instruction, frequent health evaluations, and feedback systems.
- Extending effective interventions to additional areas facing comparable issues, while tailoring strategies to local conditions.
- Creating increasingly complex models to forecast and comprehend the effects of climate change on health, enabling more focused and efficient actions.
- Finding and filling in the gaps in the community’s access to healthcare infrastructure and other critical services with an impact on health outcomes.
- Promoting laws that combine community development, health, and climate adaptation to guarantee a comprehensive strategy for long-term health gains.
- Focusing on maternal and child health can empower community members with knowledge about ANC, nutrition, and safe childbirth practices, leading to improved health outcomes.
- Establishing integrated healthcare services that combine maternal health with nutrition and food security initiatives will enhance access to essential care and promote resilience against climate-related challenges.

## Conclusion

Persuading the community members to embrace change was a challenging task, as parents feared sending their children away, while elders were concerned about preserving their customs. However, the NGO emphasized the importance of independence and the benefits of having locally trained medical professionals, which led to children returning to establish clinics and educate their peers on preventative measures against illnesses related to climate change. Consequently, Kulawa, once a remote community lacking healthcare facilities and personnel, emerged as a model of resilience and survival. The health knowledge gained by the trained children fostered a deep sense of responsibility and care for their community, prompting initiatives to continue educating future generations.

Kulawa’s transformation illustrates the power of community-driven change and education, highlighting the significance of nurturing human potential in challenging circumstances. By focusing on healthcare education, Kulawa significantly improved its health standards and adapted to a changing environment. Their story demonstrates that sustainable development is possible when individuals are equipped with the knowledge and resources to overcome obstacles. It underscores the importance of culturally sensitive approaches to effecting lasting change and serves as a valuable example for other hard-to-reach communities striving to safeguard their health.

The return of trained community members had a profound impact, leading to substantial improvements in the population’s health. With the knowledge they brought back, the community was able to enhance its health despite the challenges posed by annual flooding. They also adopted new health strategies that integrated traditional healing practices with modern techniques, fostering collaboration between traditional healers and trained professionals for holistic care. This journey of transformation showcases the potential of community-driven, culturally sensitive approaches to build resilience against climate change threats to health. Future initiatives can leverage the lessons learned from the Kulawa community to create healthier and more sustainable communities, addressing ongoing challenges and fostering long-term well-being.

## Summary of Key Findings/Implications

- Women were confined to their homes based on cultural practices, this isolation was intensified during floods, limiting their accessibility to distanced health services. The annual floods worsened by climate change made access to the health center hard, emphasizing the need for within-reach healthcare systems.
- The pregnant women relied on Traditional Birth Attendants, only seeking formal care during complications; annual flood incidence increased maternal mortality risks during the complications.
- Means of transportation to and from the community are largely motorcycles, worsened by the annual floods, and become impractical. The climate change effect contributes to transportation barriers in healthcare access.
- The NGO’s persistent engagement with the community facilitates trust building, an essential component of community engagement that ensures resilience and adaptation practices against climate-driven health risks.
